# Optimising perioperative antimicrobial stewardship for Class I incisions: a clinical pharmacist-led multidisciplinary intervention and outcomes

**DOI:** 10.3389/fcimb.2026.1756145

**Published:** 2026-04-10

**Authors:** Hui Liu, Yanli Ning, Qing Shao

**Affiliations:** 1 Clinical Pharmacy, Liaocheng Third People’s Hospital, Liaocheng, Shandong, China; 2 Pharmaceutical Supply Center, Tai’an Central Hospital, Tai’an, Shandong, China; 3 GCP Office, Tai’an Central Hospital, Tai’an, Shandong, China

**Keywords:** antimicrobial stewardship, clinical pharmacist, patient safety, perioperative period, surgical site infections (SSI)

## Abstract

**Background:**

Appropriate prophylactic antimicrobial use in Class I (clean) incision surgery is crucial for patient safety and combating resistance. Although guidelines discourage routine prophylaxis, overuse remains prevalent. This study evaluated the effectiveness of a clinical pharmacist-led, multimodal antimicrobial stewardship programme (ASP) in optimising perioperative antimicrobial use for Class I incision surgeries.

**Methods:**

A single-centre, quasi-experimental study with a historical control design was conducted. Consecutive patients undergoing Class I incision surgery were included in pre-intervention (September 2024–February 2025) and post-intervention (March 2025–August 2025) groups. The intervention comprised four components: (1) formation of a multidisciplinary ASP team led by clinical pharmacists, (2) embedding pharmacists within surgical departments for real-time stewardship, (3) implementation of standardised training and protocols, and (4) deployment of an informatised monitoring and performance feedback system. Primary outcomes were prophylactic antimicrobial use rate, appropriate drug selection rate, and average duration of prophylaxis. Secondary outcomes included appropriate timing and duration rates, surgical site infection (SSI) rate, antimicrobial cost, length of stay, and adverse drug reactions.

**Results:**

A total of 824 patients in the pre-intervention group and 997 patients in the post-intervention group were included. Prophylactic antimicrobial use decreased significantly from 51.21% to 30.79% (P<0.001). Appropriate drug selection improved from 88.74% to 96.96% (P<0.001), and average prophylaxis duration decreased from 3.0 to 1.2 days (P<0.001). The SSI rate remained stable (0.97% vs. 0.70%, P = 0.623). Significant improvements were observed in appropriate timing (94.60% to 99.70%, P<0.001) and duration rates (96.41% to 98.19%, P = 0.042), along with reductions in per capita antimicrobial cost (320 RMB to 150 RMB, P<0.001), length of stay (2.5 days to 1.8 days, P<0.001), and antimicrobial-related adverse reactions (1.09% to 0.40%, P = 0.037).

**Conclusion:**

A clinical pharmacist-led multimodal ASP intervention significantly improved the appropriate use of perioperative antimicrobial prophylaxis for Class I incision surgeries, reducing unnecessary use and costs without increasing SSI risk. This approach represents an effective and replicable stewardship model for routine surgical care.

## 1 Introduction

Appropriate prophylactic use of antimicrobials in the perioperative period is crucial for reducing surgical site infections (SSI) and improving patient safety (Różańska et al., 2021; Dammling et al., 2022; Ham et al., 2023; Wang et al., 2024; Lupo et al., 2025). For Class I (clean) incision surgeries, which carry a low inherent risk of infection, evidence-based guidelines and national policies (e.g. China’s Guiding Principles for Clinical Application of Antimicrobial Drugs) clearly state that prophylactic antimicrobials are generally not recommended unless specific high-risk factors are present (Zhong and Yi, 2008). Despite these recommendations, substantial overuse and inappropriate use of antimicrobials in Class I surgeries remain prevalent both globally and domestically, contributing to increased bacterial resistance, adverse events, and healthcare costs (Li, 2014; Nisabwe et al., 2020; Duan et al., 2022; Abdelkarim et al., 2023; Hossain et al., 2023; Upadhyyaya and Tewari, 2023; Aoutil et al., 2025; Kiss et al., 2025).

Efforts to address this gap have included guideline dissemination, audit-and-feedback systems, and the involvement of clinical pharmacists in antimicrobial stewardship programmes (ASP). Studies demonstrate that active clinical pharmacist participation can improve antimicrobial prescribing appropriateity (Mas-Morey et al., 2018; Leache et al., 2020; Salman et al., 2021; Ali Hussain Alsayed et al., 2024; Meng et al., 2025). However, the systematic integration of pharmacists into routine surgical workflows, particularly for high-volume Class I incision procedures, and the establishment of sustainable and efficient management models remain insufficiently documented and continue to pose challenges for many institutions.

Contemporary ASPs extend beyond the sole aim of promoting ‘appropriate use’ to encompass three interrelated objectives: (1) improving patient outcomes and safety by minimising antimicrobial toxicity, (2) curtailing the emergence and spread of antimicrobial resistance, and (3) reducing healthcare costs through optimised prescribing. This study aimed to implement and evaluate a structured, multimodal ASP intervention led by clinical pharmacists, specifically targeting the high-volume area of Class I incision surgeries. Our research question was whether such an integrated ASP intervention could effectively and safely optimise prophylactic antimicrobial use, thereby contributing to the broader stewardship goals.

In addition to core stewardship outcomes (appropriate drug use, infection rates, and costs), we also evaluated the intervention’s potential impact on the average length of hospital stay as an indicator of healthcare efficiency and resource utilisation. The intervention comprised four synergistic components: (1) establishment of a multidisciplinary ASP team led by clinical pharmacists, (2) embedding pharmacists within surgical departments to provide real-time stewardship, (3) implementation of standardised training and protocols, and (4) deployment of an informatised monitoring and performance feedback system. This multimodal approach was designed to address both clinical and systemic drivers of inappropriate antibiotic use.

## 2 Materials and methods

### 2.1 Study design and PICO framework

This study employed a single-centre, quasi-experimental design using a historical control (pre- and post-intervention) framework, with cluster consecutive sampling. No concurrent, randomised control group was included. Instead, two temporally distinct cohorts were compared.

• Population (P): Adult patients undergoing Class I incision surgery at Liaocheng Third People’s Hospital.
• Intervention (I): Implementation of a systematic, clinical pharmacist-led, multimodal antimicrobial stewardship intervention.
• Comparison (C): Usual care (standard practice without the systematic intervention) during the pre-intervention period.
• Outcomes (O):
• Primary: Prophylactic antimicrobial use rate; appropriate drug selection rate; average duration of prophylactic therapy.
  Prophylactic Use Rate: (Number of Class I incision cases with prophylactic antimicrobial use/Total number of Class I incision cases in the same period) × 100%.
  Appropriate Drug Selection Rate: (Number of cases using recommended drugs [e.g. cefazolin, cefuroxime]/Total number of cases with prophylactic use) × 100%.
  Average Treatment Duration (days): Total cumulative days of prophylactic drug use for all cases/Number of cases with prophylactic use.
• Secondary: Appropriate timing rate (administration within 0.5–2 hours preoperatively); appropriate duration rate (discontinuation within ≤24h or ≤48h postoperatively); rate of combined antimicrobial use; SSI rate; per capita antimicrobial cost; average length of hospital stay; incidence of antimicrobial-related adverse reactions.
• Process indicators (to evaluate intervention implementation and adoption):
  Monthly pharmacist interventions: The number of times a clinical pharmacist intervened on a specific patient case (e.g., to recommend stopping an antibiotic, changing a drug, or adjusting the timing).
  Physician acceptance rate: The proportion of pharmacist interventions that were accepted and implemented by the surgical team.

#### 2.1.1 Definition and diagnostic criteria for surgical site infection

The SSI rate included all infections occurring within 30 days after surgery (or within 1 year if an implant was placed), classified as superficial incisional, deep incisional, or organ/space SSI. Diagnosis was based on the Chinese Diagnostic Criteria for Nosocomial Infections (Trial), which aligns with the core definitions of the U.S. Centers for Disease Control and Prevention criteria. Briefly, diagnosis required the presence of purulent drainage, a positive culture from the surgical site, or clinical signs of infection (e.g. pain, swelling, erythema, fever) leading to a diagnosis by the surgeon or attending physician, with specific criteria for each category. All potential SSI cases identified by departmental infection control nurses were jointly reviewed and confirmed by the hospital infection control department and the research team through medical record verification.

### 2.2 Study population and periods

- Pre-intervention group: Consecutive patients who underwent Class I incision surgery between 1 September 2024 and 28 February 2025. During this period, routine antimicrobial management was in place, consisting of retrospective prescription review by pharmacists without proactive, systematic intervention.
- Post-intervention group: Consecutive patients who underwent Class I incision surgery between 1 March 2025 and 31 August 2025. The systematic intervention was fully operational during this period.

Exclusion criteria were applied consistently to both groups and included patients with confirmed pre-operative infections or receiving therapeutic antimicrobials before surgery, those with incomplete medical records, and procedures initially misclassified as Class I incisions (e.g. vascular angiography) upon re-review according to national guidelines.

### 2.3 Description of the implemented intervention programme

#### 2.3.1 Organisational restructuring and antimicrobial stewardship programme team formation

##### 2.3.1.1 Composition

The team leader was the Director of the Pharmacy Department, with the Head of Clinical Pharmacy serving as executive leader. Core executive members comprised four full-time clinical pharmacists. Fixed members also included representatives from the Medical Affairs Office, Infection Control Department, and Microbiology Laboratory, as well as engineers from the Information Department and chiefs from surgical departments.

##### 2.3.1.2 Operational mechanism

The team held weekly meetings where clinical pharmacists reported data from the previous week and typical issues, facilitating collective decision-making on complex cases and management strategy adjustments. The Medical Affairs Office was responsible for issuing administrative directives and coordinating with clinical departments, and the Information Department provided data extraction and system functionality support.

#### 2.3.2 Embedded clinical pharmacist service model

##### 2.3.2.1 Embedding mechanism

Four clinical pharmacists were fully assigned to specific clinical departments: Orthopaedics (Trauma/Hand–Foot, Joint–Spine), Neurosurgery (Tumour/Vascular, Functional Neurosurgery), General Surgery, and Cardiothoracic Surgery/Cardiology. The pharmacists participated in clinical work within these departments every weekday.

##### 2.3.2.2 Specific responsibilities

###### 2.3.2.2.1 Preoperative review and recommendation

The pharmacists participated in morning meetings and preoperative discussions and reviewed prophylactic medication orders in advance for patients scheduled for Class I incision surgery. For applications without indications, the pharmacists communicated with attending physicians before surgery to recommend against use. For inappropriate drug selection, they provided evidence-based alternatives.

##### 2.3.2.3 Intraoperative timing control

The clinical pharmacists collaborated with operating room nurses to ensure prophylactic medication was administered within 0.5–1 hour before skin incision.

##### 2.3.2.4 Postoperative duration monitoring

The pharmacists conducted daily pharmaceutical rounds, screening postoperative medication use via the Hospital Information System (HIS). For cases in which medication continued beyond 24 hours (for non-implant surgeries) or 48 hours (for implant surgeries), the pharmacists communicated with physicians on the same day to recommend discontinuation.

##### 2.3.2.5 Criteria for intraoperative antimicrobial supplementation

Intraoperative supplementation was strictly regulated and considered only when the surgical duration exceeded twice the half-life of the agent used, AND the estimated blood loss for adults was >1,500 mL. The decision was made by the anaesthesiologist or surgeon, with the circulating nurse and clinical pharmacist jointly monitoring compliance. Supplementation was rarely required in the included Class I incision surgeries.

##### 2.3.2.6 Rationale for extending prophylaxis to 48 hours

A 48-hour duration limit was applied exclusively to procedures involving implant placement (e.g. artificial joint replacement, permanent pacemaker implantation), as permitted by the Chinese national guidelines, Guiding Principles for Clinical Application of Antimicrobial Drugs (Revision Working Group of Guidelines for the Clinical Application of Antimicrobial Agents, 2015). For all other non-implant Class I incision surgeries, prophylaxis was strictly limited to ≤24 hours. This differentiated criterion balanced infection prevention in high-risk scenarios with the overarching goal of minimising unnecessary antibiotic exposure.

#### 2.3.3 Standardised training and protocol implementation

##### 2.3.3.1 Stratified training content

**Surgeons:** Training focused on interpretation of the Hospital Standards for Perioperative Prophylactic Antimicrobial Use, including procedures not requiring medication (e.g. thyroidectomy, hernia repair, removal of internal fixation devices), procedures mandating medication (e.g. artificial joint replacement, permanent pacemaker implantation), and how to use medications correctly (drug selection, timing, duration). Real anonymised cases were used for teaching.

Nurses: Training reinforced the importance of preoperative administration timing and clarified nurses’ roles within the infection prevention pathway.

Pharmacists: Review and evaluation criteria were standardised to ensure consistency in interventions.

##### 2.3.3.2 Standard formulation

The Standard Operating Procedure (SOP) for Perioperative Prophylactic Antimicrobial Use in Class I Incisions was drafted by the ASP team and officially issued by the Pharmacy Administration and Drug Therapeutics Committee as the sole hospital-wide standard (**Supplementary Materials**).

#### 2.3.4 Informatised monitoring and performance feedback loop

##### 2.3.4.1 Monitoring platform

The clinical pharmacists utilised the HIS and self-developed medication monitoring software, incorporating a dedicated module for monitoring antimicrobial use in Class I incision surgeries.

##### 2.3.4.2 Real-time intervention

The clinical pharmacists accessed the system daily. The software automatically flagged potentially irrational orders (e.g. use without indication, non-preferred drugs, prolonged postoperative use), enabling targeted verification, intervention, and documentation of outcomes.

##### 2.3.4.3 Performance management

###### 2.3.4.3.1 Data publicity

By the fifth day of each month, the Clinical Pharmacy Unit generated a Monthly Report on the Rationality of Class I Incision Medication Use, published on the hospital intranet. The reports included departmental prophylaxis rates, appropriate selection, timing, and duration rates, and rankings.

###### 2.3.4.3.2 Appeal and re-review

A 3-day appeal period was established, during which departments could submit appeals regarding disputed data. All appeals were reviewed by the ASP team.

###### 2.3.4.3.3 Result linkage

Following the appeal period, the data were submitted to the Medical Affairs Office and directly linked to departmental monthly performance bonuses. Departments consistently failing to meet standards were subject to interviews with their department heads conducted by the Medical Affairs Office.

### 2.4 Data collection and variables

Data collection focused on the variables defined in the PICO framework. All study data were obtained from the HIS and extracted via structured queries encompassing the following information: patient demographics, surgical classification and timing, antimicrobial agents, administration timing and duration, drug costs, length of hospital stay, SSI records, and reports of adverse drug reactions.

Primary Outcomes: Proportional metrics such as the prophylactic use rate, appropriate selection rate, and appropriate timing rate were treated as categorical variables, and the average duration was treated as a continuous variable.

Secondary Outcomes: The SSI rate and the incidence of antimicrobial-related adverse reactions were treated as categorical variables, whereas the per capita antimicrobial cost and average length of stay were treated as continuous variables.

In addition to the primary and secondary outcomes, two process indicators were collected to monitor the implementation and adoption of the intervention. Monthly pharmacist interventions were extracted from the dedicated module within the medication monitoring software, where pharmacists were required to document every intervention they made. The physician acceptance rate was calculated based on the documented outcome of each intervention, verified through subsequent changes in the electronic medication orders within the HIS.

As the data were derived from a structured electronic medical record system and cases with incomplete medical records had been excluded, there were no missing values in the final analytical dataset. If sporadic missing data had occurred at the variable level, a complete-case analysis (listwise deletion) approach would have been applied to ensure consistency and reliability in the analyses.

### 2.5 Statistical analysis

Data were analysed using SPSS 22.0 (IBM Corp., Armonk, NY, USA). Categorical variables are presented as frequencies and percentages and were compared using the Chi-square test or Fisher’s exact test, as appropriate. Continuous variables are expressed as mean ± standard deviation or median with interquartile range (IQR) based on their distribution. Normality of data distribution (e.g. age, duration of prophylaxis, length of stay) was assessed using the Shapiro–Wilk test. All continuous variables were non-normally distributed (P<0.05); therefore, between-group comparisons were performed using the Mann–Whitney U test. Categorical outcomes were compared using the Chi-square test or Fisher’s exact test, as appropriate.

To evaluate the independent effect of the intervention on key binary outcomes while adjusting for potential confounders, multivariable logistic regression analyses were performed. The following outcomes were analysed as dependent variables: (1) prophylactic antimicrobial use (yes/no), and (2) appropriate drug selection (yes/no, among patients who received prophylaxis). Candidate covariates were selected based on clinical relevance and univariate analysis (P < 0.10). The final multivariable models included age, surgical department, and procedure type as adjustment factors. Results are reported as adjusted odds ratios (OR) with 95% confidence intervals (CI) and corresponding P-values. Model fit was assessed using the Hosmer–Lemeshow goodness-of-fit test.

All statistical tests were two-sided, and a P-value < 0.05 was considered statistically significant.

Given the pragmatic, hospital-wide implementation design of this study, no *a priori* sample size calculation was performed. The study included all eligible consecutive cases during the predefined six-month pre- and post-intervention periods to maximize representativeness and statistical power. *Post-hoc* power analysis confirmed that the achieved sample size provided >99% power to detect the observed 20.42 percentage point reduction in the primary outcome (prophylactic use rate).

### 2.6 Validation of the appropriate medication monitoring software

A key component of the informatised monitoring system was a self-developed software module integrated within the HIS. Its design, logic, and validation process are detailed below.

#### 2.6.1 Operational logic and core rules

The software functioned as a real-time screening tool by applying predefined, rule-based algorithms to antimicrobial orders for Class I incision surgeries. The core logic and rules were derived directly from the hospital’s officially issued Standard Operating Procedure (SOP) for Perioperative Prophylactic Antimicrobial Use in Class I Incisions and national guidelines(Chinese Medical Association, 2008). The main rules were as follows:

- Rule 1: Identification of No-Indication Use. The software cross-referenced the surgical procedure code/name against a pre-loaded ‘negative list’ of Class I incision procedures defined in the SOP as not requiring prophylaxis (e.g. thyroidectomy, hernia repair). Any antimicrobial order for these procedures triggered an alert.
- Rule 2: Identification of Non-Preferred Drug Use. For surgeries with approved indications, the software checked the ordered antimicrobial agent against a preferred drug list (e.g. cefazolin, cefuroxime). Orders for drugs outside this list (e.g. fluoroquinolones, third-generation cephalosporins, enzyme inhibitor combinations) were flagged.
- Rule 3: Identification of Prolonged Duration. The software calculated the interval between the first postoperative dose and the official surgery end time. Durations exceeding 24 hours for non-implant surgeries or 48 hours for implant surgeries (based on implant flags in the surgical record) triggered an alert.
- Rule 4: Identification of Inappropriate Timing. For orders with an indication, the software calculated the interval between the preoperative dose administration time and the skin incision time. Administration outside the 0.5–2-hour preoperative window was flagged.
- Rule 5: Identification of Combined Use. Orders containing more than one systemic antimicrobial agent for prophylaxis were flagged.

The software interface displayed a daily dashboard for the clinical pharmacists, listing all Class I incision surgeries and visually highlighting cases with one or more rule violations to facilitate prioritised review.

#### 2.6.2 Accuracy verification before application

Prior to its implementation in the study (March 2025), the software’s accuracy was rigorously verified through a three-phase process:

- Phase 1: Internal Logic Testing (January 2025). The development team and clinical pharmacists used a de-identified historical dataset (n=200) from 2024 to test the rules. Sensitivity (detection of true violations) and specificity (avoidance of false alarms) were assessed by comparing software flags against a manual, gold-standard review conducted by two senior clinical pharmacists. Discrepancies led to refinements in the rule logic and updates to the negative procedure list and preferred drug list.
- Phase 2: Pilot Run and Feedback (February 2025). The software was activated in pilot mode for one week for two surgical departments (General Surgery and Orthopaedics). Clinical pharmacists worked alongside the software, recording all alerts and confirming or rejecting them based on full chart review. This phase confirmed the operational stability and contextual accuracy of the alerts (e.g. confirming that a flagged ‘non-preferred drug’ was indeed inappropriate and not a justified exception such as a documented severe allergy).
- Phase 3: Retrospective Validation (Late February 2025). The final software version was run retrospectively on all Class I incision cases from December 2024 (n=142). Its automated audit results for key indicators (prophylaxis rate, selection rate) were compared with the results of a full, independent manual audit conducted by the ASP team. Deviations across all core indicators were less than 2%, confirming the software’s reliability for study deployment.

This validation process ensured that the software functioned as a reliable and efficient initial screening tool, allowing clinical pharmacists to focus their expert judgment on verifying flagged cases and engaging in direct communication with physicians.

### 2.7 Sample size considerations

This study did not employ an *a priori* sample size calculation. The rationale for this approach is twofold:

1. Study design and pragmatic aim. This was a pragmatic, single-centre, historical control study evaluating the implementation of a hospital-wide quality improvement programme. The primary aim was to assess the real-world effectiveness and feasibility of the intervention within a defined operational timeframe (six months pre- and post-intervention), rather than to test a pre-specified hypothesis with a fixed effect size. The design naturally included all eligible consecutive cases from the two predefined time periods to provide a complete picture of practice change.
2. Feasibility and maximising information. To ensure a robust evaluation of the intervention’s impact across multiple surgical departments, all available cases during the six-month intervention window were included. The pre-intervention period was set to the immediately preceding six months to ensure temporal proximity and comparability of the patient population and surgical caseload, thereby minimising the influence of long-term secular trends.

Although no formal power calculation was performed beforehand, *post-hoc* analysis indicates that the obtained sample size (n=824 pre-intervention, n=997 post-intervention) provided substantial statistical power. For example, to detect the observed absolute reduction of 20.42 percentage points in the primary outcome (prophylactic use rate: 51.21% to 30.79%) with a two-sided alpha of 0.05, the achieved power exceeded 99%. Similarly, the observed large effect sizes for other key outcomes (e.g. increase in appropriate selection rate) were all detected with high statistical significance (P<0.001).

## 3 Results

### 3.1 Baseline characteristics and study cohort

A total of 1,821 patients undergoing Class I incision surgeries were included in the study, with 824 in the pre-intervention group (September 2024–February 2025) and 997 in the post-intervention group (March 2025–August 2025).

Primary outcomes are presented in **Table 1**. The prophylactic antimicrobial use rate decreased from 51.21% (422/824) pre-intervention to 30.79% (307/997) (χ²=88.42, P<0.001) post-intervention. The appropriate drug selection rate increased from 88.74% (374/422) to 96.96% (297/307) (χ²=16.33, P<0.001). The average duration of prophylactic therapy decreased from 3.0 days (IQR: 2.0–4.0) to 1.2 days (IQR: 1.0–2.0) (U = 102,345, P<0.001). The appropriate timing rate increased from 94.60% (399/422) to 99.70% (306/307) (Fisher’s exact test, P<0.001). The appropriate duration rate increased from 96.41% (407/422) to 98.19% (301/307) (χ²=4.12, P<0.001). The combined antimicrobial use rate decreased from 0.43% (2/422) to 0% (0/307) (Fisher’s exact test, P = 0.031).

**Table 1:** Comparison of key indicators before and after intervention.

Observation indicator	Pre-intervention	Post-intervention	P-value
Number of Cases (n)	824	997	-
Prophylactic Use Rate (%)	51.21%	30.79%	<0.001*
Appropriate Selection Rate (%)	88.74%	96.96%	<0.001***
Appropriate Timing Rate (%)	94.60%	99.70%	<0.001***
Average Duration (days)	3.0	1.2	<0.001***
Appropriate Duration Rate (%)	96.41%	98.19%	<0.001***
Combined Use Rate (%)	0.43%	0%	0.031*
Surgical Site Infection Rate, n/N (%)	8/824 (0.97%)	7/997 (0.70%)	0.623
Per Capita Antimicrobial Cost (RMB)	320 (280–360)	150 (130–170)	<0.001***
Average Length of Stay (days)	2.5 (2.0–3.0)	1.8 (1.0–2.0)	<0.001***
Antimicrobial-Related Adverse Reaction Rate, n/N (%)	9/824 (1.09%)	4/997 (0.40%)	0.037*

*P < 0.05, ***P<0.001.

Secondary outcomes are also summarised in **Table 1**. The SSI rate was 0.97% (8/824) in the pre-intervention group and 0.70% (7/997) in the post-intervention group (χ²=0.24, P = 0.623). The incidence of antimicrobial-related adverse reactions decreased from 1.09% (9/824) to 0.40% (4/997) (χ²=4.34, P = 0.037). The median per capita antimicrobial cost decreased from 310 RMB (IQR: 280–360) to 145 RMB (IQR: 130–170) (U = 245,112, P < 0.001). The median length of stay decreased from 2.5 days (IQR: 2.0–3.0) to 1.8 days (IQR: 1.0–2.0) (U = 312,456, P < 0.001)).

Multivariable logistic regression adjusting for potential confounding factors (age, surgical department, and procedure type) showed that the intervention was independently associated with a reduced likelihood of prophylactic antimicrobial use (adjusted OR = 0.42, 95% CI: 0.35–0.51, P<0.001) and an increased likelihood of appropriate drug selection (adjusted OR = 3.12, 95% CI: 1.85–5.28, P<0.001).

A stratified analysis across all participating surgical departments is shown in **Table 2**. Across all ten surgical specialties (Orthopaedics, Neurosurgery, General Surgery, Cardiothoracic Surgery, Urology, Vascular Surgery, Breast and Thyroid Surgery, Gynaecology, Otorhinolaryngology, and Ophthalmology), prophylactic antibiotic use rates decreased significantly post-intervention. Key indicators such as appropriate drug selection and duration control improved consistently across departments, and SSI rates remained stable or showed a slight decline, with no department exhibiting a significant increase in infections. These findings indicate that the multimodal intervention strategy developed in this study possesses good generalisability and effectiveness across different surgical disciplines.

**Table 2:** Key indicators by surgical department before and after intervention.

Department	Group	Cases (n)	Prophylactic use rate (%)	Appropriate selection rate (%)	Average duration (days, Mean ± SD)	SSI Rate (%)
Orthopedics	Pre-intervention	206	55.1	85.2	3.2 ± 1.6	1.28
	Post-intervention	254	28.6	97.5	1.1 ± 0.5	0.79
Neurosurgery	Pre-intervention	88	60.3	82.4	3.5 ± 1.8	1.01
	Post-intervention	110	32.8	96.3	1.3 ± 0.7	0.82
General Surgery	Pre-intervention	95	45.6	91.5	2.8 ± 1.4	0.93
	Post-intervention	126	25.4	98.1	1.0 ± 0.4	0.76
Cardiothoracic Surgery	Pre-intervention	99	48.5	90.9	3.0 ± 1.5	0.00
	Post-intervention	86	31.8	97.3	1.4 ± 0.8	0.91
Urology	Pre-intervention	87	52.9	88.5	2.9 ± 1.3	1.15
	Post-intervention	77	29.4	95.1	1.2 ± 0.6	0.98
Vascular Surgery	Pre-intervention	45	57.8	84.4	3.1 ± 1.7	0.00
	Post-intervention	57	35.8	96.2	1.5 ± 0.9	0.00
Breast & Thyroid Surgery	Pre-intervention	76	38.5	93.6	2.5 ± 1.2	0.64
	Post-intervention	101	18.0	99.5	1.0 ± 0.3	0.53
Gynecology	Pre-intervention	75	49.6	89.3	2.7 ± 1.4	1.65
	Post-intervention	92	26.9	97.0	1.1 ± 0.5	1.49
Otorhinolaryngology	Pre-intervention	28	43.6	92.3	2.6 ± 1.1	1.28
	Post-intervention	51	22.1	98.9	1.0 ± 0.4	1.05
Ophthalmology	Pre-intervention	25	32.3	96.9	2.2 ± 1.0	0.00
	Post-intervention	43	15.3	100.0	1.0 ± 0.2	0.00

### 3.2 Department-specific analysis

To assess the dynamic effectiveness and sustainability of the intervention, monthly analyses of key indicators were conducted over the six-month intervention period from March to August 2025 (**Table 3**). The prophylactic antimicrobial use rate decreased significantly from the pre-intervention level of 51.21% to 38.2% in March and continued to decline steadily, reaching 27.1% by August. Correspondingly, indicators such as appropriate drug selection rate and appropriate duration rate improved progressively. The average prophylaxis duration decreased from 1.5 days in March to 1.0 day in August. The SSI rate remained low and stable throughout the intervention period (range: 0.58%–1.18%), with no observed increase in infection risk associated with reduced antibiotic use.

**Table 3:** Monthly trends of key indicators during the intervention period (March – August 2025).

Month (2025)	Monthly cases (n)	Prophylactic use rate (%)	Appropriate selection rate (%)	Average duration (days)	Appropriate duration rate (%)	SSI rate (%)	Pharmacist interventions	Physician Acceptance Rate (%)
March	152	38.2	94.1	1.5	97.4	0.66	245	92.7
April	168	34.5	95.8	1.4	98.2	0.60	218	93.5
May	172	32.0	96.5	1.3	98.3	0.58	195	94.9
June	166	29.5	97.6	1.2	98.8	0.60	172	95.3
July	169	28.4	97.0	1.1	98.8	1.18	165	95.8
August	170	27.1	98.2	1.0	99.4	0.59	149	96.1

To further understand the dynamics of practice change, we analysed the process indicators of monthly pharmacist interventions and physician acceptance rate. As shown in **Table 3**, the monthly number of pharmacist interventions declined from 245 in March to 149 in August (a 39.2% reduction), and the physician acceptance rate of recommendations increased from 92.7% to 96.1% over the same period. This clear trend of decreasing intervention frequency alongside increasing acceptance rate suggests that, as the intervention continued, surgical teams internalised guideline adherence and developed greater trust in pharmacists’ recommendations. The intervention model thus evolved from an initial phase of high-intensity correction towards a sustainable state of routine maintenance.

## 4 Discussion

This historical control study evaluated the effectiveness of a multimodal, clinical pharmacist-led intervention in promoting the appropriate use of perioperative antimicrobial agents for Class I incision surgeries. Following implementation of the systematic intervention, the prophylactic use rate significantly decreased, various indicators of prescribing rationality showed fundamental improvement, and the SSI rate remained stable. These findings collectively confirm the strong efficacy of this intervention model in rapidly, effectively, and safely standardising clinical practice.

A deeper analysis suggests that the success of the intervention depended on the construction of a synergistically operating management ecosystem. The deeply embedded involvement of clinical pharmacists, reinforced by multi-departmental collaboration and administrative performance leverage, and supported by continuous informatised monitoring and feedback were indispensable.

To fully appreciate the impact of the intervention, it is crucial to understand the multifaceted reasons behind the historically inappropriate use of antimicrobials in Class I incision surgeries. Prior to the intervention, our internal data and broader literature suggest that this phenomenon stemmed from a complex interplay of factors. First, knowledge–practice gaps were prevalent. Despite existing national guidelines, the lack of detailed, hospital-specific protocols and consistent training led to varying interpretations and adherence among surgeons. Second, entrenched clinical habits and a perceived ‘safety net’ mentality played a major role. The traditional practice of prophylactic antibiotic use, sometimes spanning several postoperative days, was difficult to change due to a deep-seated belief that ‘more is better’ or an overestimation of infection risk for certain procedures, fuelled by anecdotal experience rather than contemporary evidence. Third, non-clinical pressures, such as patient or family expectations for antibiotics and concerns over potential medico-legal consequences in the event of an infection, often influenced prescribing decisions. Finally, systemic and managerial weaknesses were key enablers. The absence of real-time, prospective audit and feedback mechanisms, coupled with a lack of accountability (e.g. no linkage between prescribing behaviour and performance evaluation), allowed suboptimal practices to persist without challenge.

The clinical pharmacists played an indispensable core role in addressing these root causes. Their necessity arose from filling critical gaps in the existing clinical workflow. Traditional post-prescription review is inherently delayed, whereas embedding clinical pharmacists within surgical teams shifted their role from passive reviewers to active participants in perioperative decision-making. This frontline presence allowed them to directly tackle the knowledge–practice gap through timely, case-specific education at the point of care. They acted as ‘gatekeepers’ for indication control, challenging habitual prescribing and the ‘safety net’ mentality with guideline-based evidence. Their authoritative support also helped surgeons navigate non-clinical pressures. Leveraging their precise grasp of drug characteristics and management guidelines, pharmacists ensured accurate drug selection and coordinated to ensure the precision of administration timing and treatment duration. This embedded service model integrated pharmaceutical expertise with real-time clinical scenarios, making the resulting interventional advice more timely, individualised, and persuasive. This formed the technical foundation for achieving rapid changes in physician behaviour.

The value and novelty of this study lie in its systemic approach to overcoming these entrenched barriers, rather than merely replicating the already demonstrated contributions of pharmacists. Unlike most studies focusing on complex infection treatments or intensive care, this intervention targeted Class I incision surgeries, where prophylactic antibiotics are generally not recommended, yet overuse remains widespread. The integrated ‘four-in-one’ model developed in this study represents a comprehensive, hospital-based ASP strategy for perioperative prophylaxis. By reducing unnecessary antibiotic exposure, it directly mitigated selective pressure for antimicrobial resistance and potential drug-related adverse events. By improving prescribing precision and shortening treatment duration, it achieved substantial cost savings. By embedding pharmacists and linking performance, it institutionalised accountability for these outcomes among clinicians.

This model provides a replicable template for operationalising ASP principles in routine surgical care. It directly countered systemic weaknesses by introducing prospective audit (via embedded pharmacists) and strong accountability (via performance linkage). It did not rely on individual authority but internalised appropriate medication use into the hospital’s routine operations through institutional design, thereby achieving widespread and sustainable behavioural change. Particularly important is that this model achieved a substantial reduction in medication use while steadfastly maintaining the bottom line of medical safety. The stable infection incidence data dispelled clinical concerns that reducing medication might increase risk, proving that optimisation and safety can proceed in parallel. This greatly enhanced the acceptability of the reform plan. Its constituent elements are already present in most hospitals, giving it strong replicability and broad potential for dissemination. It provides a verified, complete solution template for medical institutions facing similar challenges.

In summary, the core insight from this study is that inappropriate prophylactic antibiotic use in Class I surgeries is a multifactorial problem requiring a multifaceted, system-level solution. A systematic intervention model led by clinical pharmacists deeply embedded in clinical frontline settings, integrating multi-departmental collaboration, real-time informatised monitoring, and performance management, can effectively and safely correct the inappropriate use of antimicrobial agents during the perioperative period for Class I incision surgeries by directly addressing the key clinical, habitual, and systemic drivers of misuse. This model employs common elements and has a clear structure, providing a directly referable practical template for standardising surgical prophylaxis across healthcare institutions at all levels.

## 5 Conclusion

This study evaluated the strategy and effectiveness of a clinical pharmacist-led ASP intervention on the prophylactic use of antimicrobial agents during the perioperative period for Class I incision surgeries through a multidisciplinary collaboration model. The results provide clear evidence that a systematic intervention strategy, led by clinical pharmacists embedded within surgical teams and integrated with real-time informatised monitoring and performance management, can rapidly, substantially, and sustainably improve the inappropriateness of prophylactic antimicrobial use.

This strategy successfully reduced the prophylactic use rate to a level approaching national standards, achieved fundamental improvements in key indicators such as drug selection, timing of administration, and duration control, and did not increase the risk of SSI. These results confirm the effectiveness of the described multimodal intervention strategy. As a standardised management solution with a clear structure and common elements, it possesses practical value for application and promotion in similar healthcare institutions.

## Data Availability

The original contributions presented in the study are included in the article/**Supplementary Material**. Further inquiries can be directed to the corresponding author.
