# Supplementary material for: Optimising perioperative antimicrobial stewardship for Class I incisions: a clinical pharmacist-led multidisciplinary intervention and outcomes
Source: Front Cell Infect Microbiol. 2026 Apr 10;16:1756145. doi: 10.3389/fcimb.2026.1756145 (PMC13105934; doi:10.3389/fcimb.2026.1756145)
Supplement: Supplementary file 1 [file Table1.docx]

**Standard Operating Procedure (SOP) for Perioperative Prophylactic Antimicrobial Use in Class I Incisions**

1. Multidisciplinary Collaboration Team Building

1.1 Deployment of Clinical Pharmacists to Surgical Departments

- Four clinical pharmacists will be assigned to specific surgical departments:
  - One (1) to Orthopaedics
  - One (1) to Neurosurgery
  - One (1) to General Surgery
  - One (1) to Thoracic Surgery
- Responsibilities include active participation in pre-operative discussions, post-operative ward rounds, and the formulation of medication regimens.

1.2 Establishment of an Antimicrobial Stewardship Programme (ASP) Management Team

- The ASP team will comprise members from the Intensive Care Unit (physician), Microbiology Laboratory, Medical Affairs Department, and Information Technology Department.
- The team will convene regular meetings to analyse data and optimise antimicrobial management strategies.

2. Precision Training and Standardisation

2.1 Tiered Training Programmes

- For Surgeons: Training will focus on the indications for prophylactic use in Class I Incision surgeries, appropriate antibiotic selection, and duration control.
- For Nursing Staff: Training will emphasise the importance of pre-operative antibiotic administration timing, pre-operative skin preparation, and aseptic techniques in reducing SSI, thereby reducing reliance on antimicrobials.
- For Pharmacists: Training will enhance their ability to review medication orders, emphasising key audit points. A review standard will be established based on the Clinical Application Guidelines for Antimicrobial Agents.

2.2 Reinforcement of Hospital Perioperative Antimicrobial Prophylaxis Guidelines by the Pharmacy Committee

- Clearly define Class I Incision procedures that do not require prophylaxis (e.g. thyroidectomy, breast surgery, herniorrhaphy).
- Specify high-risk factors mandating prophylaxis (e.g. implant placement, diabetes, immunocompromised status).
- Recommend first-line agents: a single dose of cefazolin or cefuroxime.
  - Avoid the use of broad-spectrum antibiotics such as fluoroquinolones and third-generation cephalosporins.
  - For patients with a history of antimicrobial allergy, especially to β-lactams (The Revision Working Group of the Guidelines for Clinical Application of Antimicrobial Agents, 2015; Ban et al., 2017):
    - For gram-positive coverage, clindamycin is the primary alternative. Its efficacy in preventing SSI has been validated in studies of orthopaedic and cardiac surgeries.
    - For gram-negative coverage (if required by the procedure), aztreonam (a monobactam) can be used, noting its narrow spectrum. Aminoglycosides (e.g. gentamicin) are another option but require caution due to potential nephro- and ototoxicity.
    - Vancomycin is reserved only for patients with a documented history of MRSA colonisation/infection or in institutions with a high prevalence of MRSA infections.
  - The goal is to use the narrowest effective spectrum and avoid unnecessary combination therapy. Clinical pharmacists provide real-time, case-specific recommendations based on this protocol during preoperative review.

3. Real-Time IT Monitoring and Intervention

3.1 Daily Pharmacy Ward Rounds and Feedback

- Clinical pharmacists will conduct daily screenings of medication orders for surgical patients via the hospital information system.
- Cases of irrational antimicrobial use will be addressed through immediate communication and formal feedback (via phone or written report).

3.2 Establishment of a Usage Rationality Notification System and Integration into Performance Management

- Monthly data on prophylactic antibiotic usage rates, appropriateness rates, and average treatment duration for each surgical department will be published.
- A 3-day appeal period will follow data publication to foster transparent and constructive competition.
- Departments with irrational medication practices will be subject to score deductions according to the hospital’s performance evaluation standards.

**Reference**

Guidelines for the Clinical Application of Antimicrobial Agents Revision Working Group. (2015). Guidelines for the Clinical Application of Antimicrobial Agents: 2015 Edition.

Ban, K.A., Minei, J.P., Laronga, C., Harbrecht, B.G., Jensen, E.H., Fry, D.E., et al. (2017). American College of Surgeons and Surgical Infection Society: Surgical Site Infection Guidelines, 2016 Update. Journal of the American College of Surgeons, 224(1), 59–74. https://doi.org/10.1016/j.jamcollsurg.2016.10.029
